# Integrated environmental assessment of plastic pollution in the São Francisco River estuary, Brazil

**DOI:** 10.1007/s10661-026-16107-1

**Published:** 2026-10-29

**Authors:** Isabella Ferreira Nascimento Maynard, Pamela Cunha Bortoluzzi, Thigna Carvalho Batista, Rosa Cecília Santos, Juliana Cordeiro Cardoso, Rubens Riscala Madi, Eliane Bezerra Cavalcanti, Verônica de Lourdes Sierpe Jeraldo, Maria Nogueira Marques

**Affiliations:** 1 Postgraduate Program in Process Engineering, Tiradentes University, 300 Murilo Dantas Avenue, Farolândia, Aracaju, Sergipe Brazil; 2 Postgraduation Program in Chemical Engineering, Federal University of Ceará, 2853 Universidade Avenue, Benfica, Fortaleza, Ceará Brazil; 3 Postgraduate Program in Biosciences and Health, Tiradentes University, Aracaju, Sergipe Brazil; 4 São Francisco River Basin Committee, Aracaju, SE Brazil; 5 Institute of Technology and Research (ITP), 300 Murilo Dantas Avenue, Farolândia, Aracaju, SE Brazil

**Keywords:** Bisphenol A, Dibutyl phthalate, Environmental monitoring, Microplastics, Macroplastics, Plasticizers

## Abstract

Plastic pollution has emerged as a major environmental concern due to its persistence, fragmentation, and capacity to release chemical additives into aquatic systems. Estuaries are particularly vulnerable, functioning simultaneously as accumulation zones and transfer pathways for plastic debris and associated contaminants. This study provides a combined assessment of macroplastics, microplastics, and plasticizers (bisphenol A – BPA and dibutyl phthalate – DBP) in the São Francisco River estuary, northeastern Brazil. Surface water and sandy sediment samples were collected at four sites, including urban areas and locations near Conservation Units. Plastic debris was quantified and characterized, while BPA and DBP were determined as chemical markers of plastic pollution. BPA was detected in 100% of water samples and DBP in 67%, with the highest concentrations recorded in the urbanized site of Propriá (BPA: 0.72 mg · L⁻^1^; DBP: 2.27 mg · L⁻^1^). The highest quantities of microplastics in the water were recorded in Santana de São Francisco (0.49 items/m^3^) and in the sandy sediment in Pacatuba (22,80 items/m^2^), with a predominance of white fragments. Macroplastics showed a progressive increase in Pacatuba, totaling 2457 g. The results highlight urban influence and the estuary’s role as a retention and transfer zone for plastic pollutants. The study employed multiple methodologies to analyze plastic pollution across scales, providing novel data for environmental monitoring in estuaries and Conservation Units. Findings reinforce the need for policies to reduce plastic use, improve sanitation, and promote environmental education, contributing to Sustainable Development Goals 6, 12, and 14.

## Highlights

• First combined assessment of macroplastics, microplastics, BPA, and DBP in the São Francisco estuary.

• Multiscale analysis in surface waters and sandy sediments.

• BPA was detected in 100% of water samples and DBP in 67% of them.

• Higher concentrations of plasticizers were recorded near urban areas.

• White microplastic fragments predominated in both water and sediments.

• International plastic bottles were found on a beach within a Conservation Unit.

**Graphical abstract:**

## Graphical abstract

**Figure:**
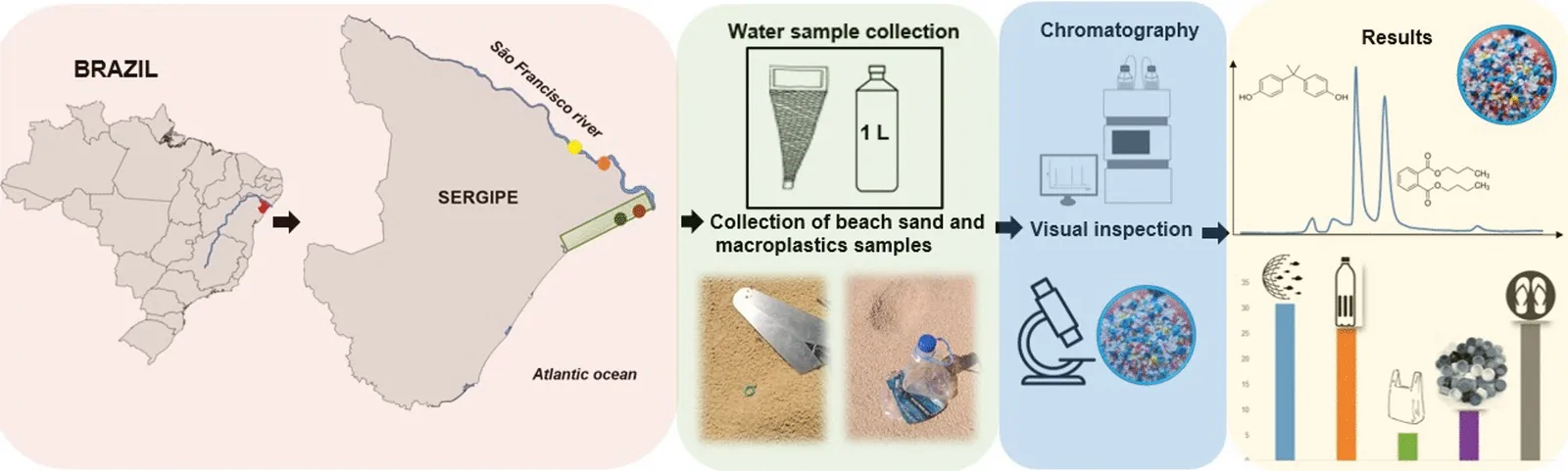

## Introduction

Chemicals and waste are part of everyday life in the modern world, and their improper management can result in impacts on human health and environmental degradation. Global annual plastic production exceeds 359 million tons, and projections indicate that, if current trends continue, up to 12 billion tons of plastic waste could accumulate in the environment by 2050, highlighting the growing challenge of solid waste management on a global scale (UNEP, 2024a). Plastics account for a significant portion of municipal solid waste, often ranking third in abundance after paper and organic waste (Fayshal, 2024). Their widespread use in sectors such as packaging, construction, electronics, and household appliances, combined with their durability and low cost, contributes to their persistent accumulation in the environment (Plastics Europe, 2016; UNEP, 2024a, b). In the environment, plastic waste undergoes mechanical and chemical deterioration, fragmenting into progressively smaller particles; particles smaller than 5 mm are defined as microplastics (Andrady, 2015; Arthur et al., 2009; GESAMP, 2015; Thompson et al., 2004).

Among the chemical compounds used in the production of various types of plastics, bisphenol A (BPA) and dibutyl phthalate (DBP) stand out. In this study, both are described as markers of plastic pollution. In addition, these compounds are recognized as endocrine disruptors, capable of interfering with the hormonal system of organisms, affecting reproductive, metabolic, and developmental functions (Maradonna et al., 2022). For this reason, they are classified among the substances prioritized for environmental monitoring, according to a list published by the Danish Environmental Protection Agency (Danish EPA, 2018; Maradonna et al., 2022).

International studies, including those by Gil-Solsona et al. (2022) and Yang et al. (2024), have adopted integrated approaches to assess plastic pollution through the combined analysis of macroplastics/microplastics and chemical contaminants derived from plastics. However, similar studies are still scarce in Brazilian ecosystems, particularly in estuarine environments of Northeast Brazil, where data on the occurrence and interaction of these pollutants are still limited (Santos et al., 2026; Trindade et al., 2023).

Most research has concentrated on marine systems, resulting in significant knowledge gaps regarding the occurrence, distribution, and impacts of microplastics and plasticizers in Brazilian rivers and estuaries (Souza et al., 2023; Trindade et al., 2023). Estuaries, as transitional zones between continental and marine environments, exhibit high ecological relevance and support essential socioeconomic activities such as fisheries, tourism, and water supply (Miller, 2013; Leal et al., 2022). However, their proximity to urban, industrial, and agricultural areas increases their vulnerability to the input of plastic debris and associated contaminants, including plasticizers (Frena et al., 2016; Ivar do Sul et al., 2014, Trindade et al., 2023).

The São Francisco River plays a strategic role in Brazil’s territorial integration and socioeconomic development. Extending approximately 2700 km, its drainage basin encompasses seven states and nearly 8% of the national territory, establishing an important hydrological connection between the Southeast and Northeast regions (CBHSF, 2025). The river supports multiple water uses, including public supply, irrigation, hydroelectric power generation, fisheries, and navigation, while simultaneously maintaining essential ecological functions across the biomes it traverses (ANA, 2022).

At its downstream limit, the São Francisco River estuary, situated between the states of Alagoas and Sergipe, constitutes a highly productive and ecologically significant coastal system. However, similar to other estuarine environments, it has been progressively subjected to anthropogenic pressures (Lima et al., 2022), notably the influx of plastic debris originating from urban, agricultural, and industrial activities throughout the watershed. In this context, systematic investigations designed to characterize and quantify plastic pollution at multiple spatial scales are critical to elucidating the magnitude, sources, and complexity of this environmental issue (PNUMA, 2021).

However, despite the recognized ecological and socioeconomic importance of the São Francisco River estuary, there are still few integrated studies on plastic pollution in this environment. Therefore, this study aims to (i) quantify and characterize macroplastics and microplastics, and (ii) identify markers of plastic pollution with potential endocrine-disrupting activity in different sectors of the estuary, including areas adjacent to Conservation Units and urbanized regions. By adopting an integrated and multi-compartment approach, this research seeks to provide a comprehensive baseline of plastic contamination in the São Francisco River estuary. The study is aligned with Sustainable Development Goals (SDGs) 6, 12, and 14 and represents the first integrated environmental assessment of plastic pollution in this estuarine system (Kumar et al., 2020, 2021).

## Methodology

### Study area

**Fig. 1:**
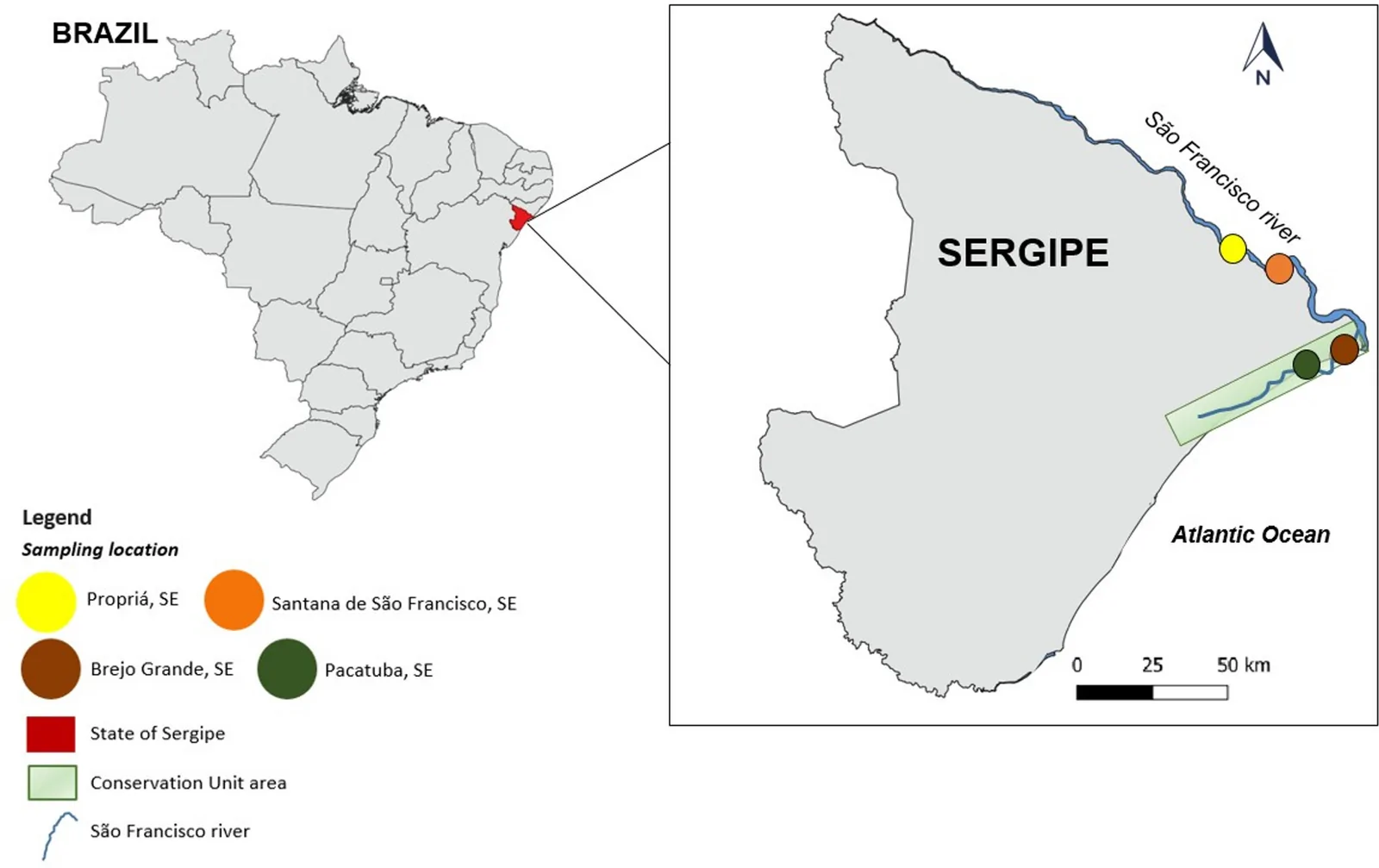
Location map of the state of Sergipe, Brazil. The colored dots identify the cities where sampling was carried out in the São Francisco River estuary

The research was conducted in northeastern Brazil, in four municipalities in the state of Sergipe, located at the mouth of the São Francisco River: Brejo Grande (WGS 84, UTM 0774893; 8,834,458), Pacatuba (WGS 84, UTM 0766083; 8,831,891), Santana de São (WGS 84 UTM 0759784; 8,865,533) and Propriá (WGS 84 UTM 0738905; 8,870,269), as shown in Fig. 1.

### Methodological procedures

#### Determination of plastic pollution markers in surface water

The occurrence of bisphenol A and dibutyl phthalate in the surface water of the São Francisco River and its tributary, the Parapuca River, was evaluated. An Agilent Technologies 1260 Infinity II high-pressure liquid chromatograph (UPLC) with a UV–Vis/DAD detector equipped with two high-pressure pumps — G7104C 1260 Flexible Pump, and a 1260 Vialsampler automatic sampler — G7129C was used. The column used was C18 Phenomenex—Kinetex EVO 100 A (5.0 µm × 150.0 mm × 4.6 mm), with an oven temperature of 40 °C. The sample injection volume was 20 µL, mobile phase of water and methanol, gradient mode, flow rate of 1.0 mL/min, and run time of 16 min, adapted from Moreira et al. (2011). Bisphenol A and dibutyl phthalate standards were injected in triplicate to construct the analytical curve at the following concentrations: 0.01; 0.05; 0.10; 0.25; 0.5; 0.75; 1.0; 2.5; 5.0; 7.5; 10.0 mg/L.

### Determination of microplastics in surface water

The sampling and sample preparation procedures used in this study were adapted from the National Oceanic and Atmospheric Administration (NOAA) (MASURA, 2015). Microplastic samples in water were collected at all sampling points on the São Francisco River in Propriá and Santana de São Francisco, and from the Parapuca Canal in Brejo Grande and Pacatuba, between 2018 and 2019 (*N* = 9).

The sampling method used for this study was horizontal trawling in shallow waters. The trawls were carried out using local fishing boats, which were moved at low speed. During collection, a horizontal dragnet made of commercial voile fabric (0.3 m in diameter, 1.0 m in length) was used; the pore size was not determined.

After collection, the net was washed with distilled water in the laboratory and the solid material collected was transferred to a stainless steel sieve (100-µm mesh) for wet sieving. Solids larger than 5 mm were discarded. Solids smaller than 5 mm were transferred to previously cleaned, dried, and weighed beakers and placed in an oven (Fanem) at 60 °C to dry the solid material.

After drying, the material was weighed on an analytical balance (0.01 mg precision) to determine the mass. Then, the oxidative process was carried out to degrade the organic matter. Initially, 20 mL of a 0.005 M aqueous solution of Fe (II) sulfate, acting as a reaction catalyst, were added to the beaker containing the dried residue; then, 20 mL of 35% hydrogen peroxide were added. The reaction mixture was left to stand at room temperature for five minutes. Subsequently, a magnetic stirring bar was introduced into the beaker, and the system was placed on a heating plate at 60 °C, covered with a watch glass. After the heating period, if there was still any remaining organic material, it is recommended to add another 20 mL of hydrogen peroxide. After this step, the sample was sieved again, washed, and left to dry, covered with aluminum foil (Masura et al., 2015; Olivatto, 2017).

In the density separation step, 1 L of saturated NaCl solution (density ≈ 1.2 g/cm^3^) was added. All material retained on the sieve was transferred to a separation funnel using a metal spatula and rinsed with the same NaCl solution. The funnel was covered with aluminum foil and left undisturbed for approximately 10 h. After settling, both the supernatant (containing low-density particles, including potential microplastics) and the settled fraction were visually inspected. It is important to note that this method preferentially isolates polymers with densities lower than 1.2 g/cm^3^, while higher-density polymers may remain in the non-floating fraction. Subsequently, the final sample is weighed (mass of possible microplastics) and sent for analysis.

### Determination of microplastics in beach sand

The sampling and sample preparation procedures were adapted from the European Commission (EC, 2013), the National Oceanic and Atmospheric Administration (Masura et al., 2015), Löder and Gerdts (2015), the Joint Group of Experts on the Scientific Aspects of Marine Environmental Protection (GESAMP, 2015), and the United Nations Environment Programme (UNEP, 2016).

Microplastic samples in the sand were collected from two beach strips between the river (Parapura Canal) and the sea (Atlantic Ocean): one in the municipality of Pacatuba (Ponta dos Mangues Beach) and the other in the municipality of Brejo Grande (Carapitanga Beach). The sampling protocol consisted of demarcating an area of 100 m × 20 m, where samples were collected at a depth of approximately 5 cm with a metal spoon in 50 cm × 50 cm quadrants (EC, 2013). Ten replicates were performed, and each replicate was collected at a distance of at least five meters (Maynard et al., 2021).

The collected samples were mixed, quartered for standardization to approximately 500 g, and sent for analysis in the laboratory; initially, they were dried in an oven at 60 °C and weighed. After this step, they underwent density separation. In this step, a solution of sodium chloride (density equal to 1.2 g/mL) and zinc chloride (density equal to 1.5 g/mL) was used in a beaker containing the sample. It was stirred with a glass rod, which was kept covered with aluminum foil for the sand particles to settle. Potential microplastic particles that accumulated on the surface of the zinc chloride solution were transferred to the sieve, where they were washed with distilled water. Thus, the possible microplastic particles were dried and weighed. The zinc chloride was filtered for reuse in future density separations for financial and environmental reasons (Löder and Gerdts, 2015; Maynard et al., 2021).

For the visual inspection and screening procedure, a Nikon SMZ800 stereoscopic microscope was used, images were recorded for application of the Axio vision LE64 digital processing software, and morphological analysis of the fragments was performed.

### Inventory of macroplastics in the sand of the São Francisco River estuary beach

The inventory of macroplastics (> 5 mm) in beach sand was carried out in accordance with the procedure described by OSPAR (2010) and consisted of quantifying and qualifying the plastic waste found in an area of 2000 m^2^ (the same area as the microplastic collection) on the coastal strip of the estuary. The percentage prevalence (P%) of each plastic debris category was calculated by dividing the mass of the respective category (g) by the total mass of all collected debris (g), and multiplying the result by 100. Photographic records of the waste were taken to identify the material collected. After quantification and qualification of the waste, it was sent to the selective collection of Conduta Consciente at Tiradentes University.

### Quality control of analyses

Quality assurance and quality control (QA/QC) procedures were implemented throughout all analytical stages to minimize contamination and ensure the reliability of the results. Standards of bisphenol A (BPA), dibutyl phthalate (DBP), and microplastics were employed. Procedural blanks were prepared and analyzed in parallel with the samples to verify potential contamination introduced during sample preparation, extraction, and analysis steps. To assess analytical performance, secondary recovery controls were conducted by spiking known amounts of the analytes into the matrices in order to monitor method efficiency.

For microplastic analysis, preventive contamination control measures were adopted, including the use of previously cleaned glassware, cotton laboratory clothing, and analyses conducted in a controlled environment. The results obtained from quality control procedures were considered satisfactory, indicating adequate analytical precision and reliability.

To ensure reliable results in the determination of plastic pollution markers (BPA and DBP), microplastics in water and sand, and macroplastics along the beach strandline, quality control (QC) measures were applied throughout the entire analytical process. Glass or metal materials (e.g., bottles, sieves, spoons, tweezers) were prioritized, and the use of plastic materials was avoided whenever possible, as recommended to reduce background contamination from microplastics and plastic additives. Samples, solutions, and equipment were kept covered with glass lids or aluminum foil during collection, transport, preparation, and analysis to minimize airborne particle deposition. Solutions (water, NaCl, ZnCl_2_) were previously filtered to remove suspended particles (Brander et al., 2020; Miller et al., 2020; Prata et al., 2021).

Analytical calibration curves for BPA and DBP were constructed using multiple concentration levels with triplicate injections to evaluate linearity, precision, and limits of detection, in accordance with recommended validation procedures for trace contaminants.

## Results and discussions

### Determination of plastic pollution markers in surface water

The analytical method for chromatographic determination was developed through the injection of certified analytical standards, enabling the construction of calibration curves for bisphenol A (BPA) and dibutyl phthalate (DBP). The resulting linear regression equations were y = 2.678x − 0.0462 for BPA and y = 0.8448x + 0.6174 for DBP. The method demonstrated excellent linearity, with correlation coefficients (*R*^2^) of 0.9996 for BPA and 0.9941 for DBP. The limit of detection (LOD) was 0.01 mg L⁻^1^ and the limit of quantification (LOQ) was 0.05 mg L⁻^1^ for both compounds. Overall, the analytical performance parameters indicate that the method is suitable for the reliable identification and quantification of these analytes.

**Fig. 2:**
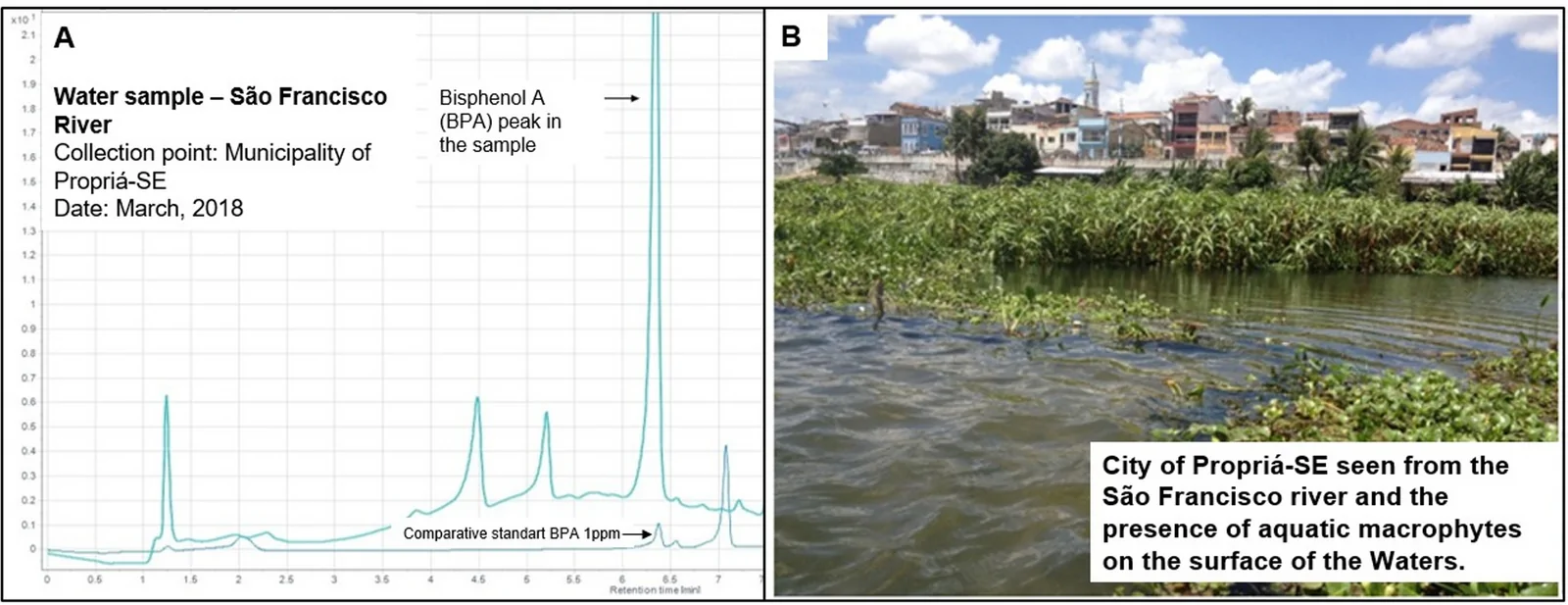
**A** Chromatogram of the preconcentrated sample from the municipality of Propriá compared to the 1 ppm mix standard (caffeine, bisphenol A, diethyl phthalate, ethinyl estradiol, dibutyl phthalate, and nonylphenol). **B** Collection point in the municipality of Propriá, near a domestic wastewater discharge point

Thus, the analyses of 12 surface water samples detected bisphenol A (BPA) in all samples, with the highest concentration in Propriá (0.72 mg · L⁻^1^, Fig. 2), near a sewage discharge point. Dibutyl phthalate was also identified in all samples, with maximum concentrations in Propriá (2.28 mg · L⁻^1^) and Santana de São Francisco (2.00 mg · L⁻^1^). It is important to note that the São Francisco River is an important source of water for public supply, especially at the collection point located in Propriá, an area subject to anthropogenic pressures, such as the improper disposal of solid waste and effluents on the riverbanks. On the other hand, in the municipalities of Pacatuba and Brejo Grande, contaminants were detected below the limit of quantification. These points are located in rural areas within the Santa Isabel Biological Reserve, which highlights the concern about the presence of pollutants even in environmental protection zones.

In Brazil, phthalates have been identified in sediments from the coastal region of Rio de Janeiro (Neves et al., 2023), thus confirming the need to monitor the quality of water bodies and their surroundings in the country. These findings corroborate previous studies that point to the widespread presence of emerging contaminants in Brazilian waters. Otomo (2015) identified BPA and dibutyl phthalate in the waters of the Guarapiranga Reservoir, São Paulo, and Machado et al. (2016) detected emerging compounds, including bisphenol A, in raw and drinking water in urban areas. Maynard et al. (2019) also reported the significant presence of plasticizers, including diethyl phthalate, dibutyl phthalate, and bisphenol A, in water supply systems in northeastern Brazil, reinforcing the persistence and spread of these contaminants in different aquatic environments.

### Determination of microplastics in river water and beach sand

#### Microplastics in surface water

Between 2018 and 2019, nine sampling campaigns were conducted using horizontal trawls at four different locations in the São Francisco River estuary, in the state of Sergipe: two sites in the Parapuca River (or Parapuca Channel), in the municipalities of Brejo Grande and Pacatuba; and two sites in the São Francisco River, in the municipalities of Propriá and Santana do São Francisco.

The sampled volume was estimated at approximately 212 m^3^ for each sampling site. The calculation considered a net with a mouth diameter of 0.30 m (radius of 0.15 m) and a towing distance of 3000 m. The net mouth area (A = πr^2^) was approximately 0.0707 m^2^, and the filtered volume (V = A × distance) was therefore estimated at approximately 212.1 m^3^.

The quantification of possible microplastics in the surface water of the São Francisco River estuary between 2018 and 2019 revealed variation between the sampling points. In Brejo Grande, concentrations were 0.23 items/m^3^ (August 25, 2018) and 0.20 items/m^3^ (August 6, 2019), with counts of 50 and 44 items, respectively. In Pacatuba, lower concentrations were recorded, reaching 0.13 items/m^3^ on June 20, 2018, and 0.08 items/m^3^ on May 21, 2019, with particle counts of 28 and 17 items, respectively.

Propriá recorded 104 items in January 2019, 72 items in August 2018, and 3 items in August 2019. Regarding concentrations, values of 0.34 items/m^3^ were observed in August 2018, 0.49 items/m^3^ in January 2019, and 0.03 items/m^3^ in August 2019. Santana de São Francisco, in turn, presented the highest absolute particle count, with 136 items recorded on January 31, 2019, and 32 items in August 2019. Concentrations at this site reached 0.64 items/m^3^ in January 2019. These results show spatial and temporal differences in the occurrence of microplastics in the estuary, with locations and periods showing greater fragmentation or greater accumulation of particles.

**Fig. 3:**
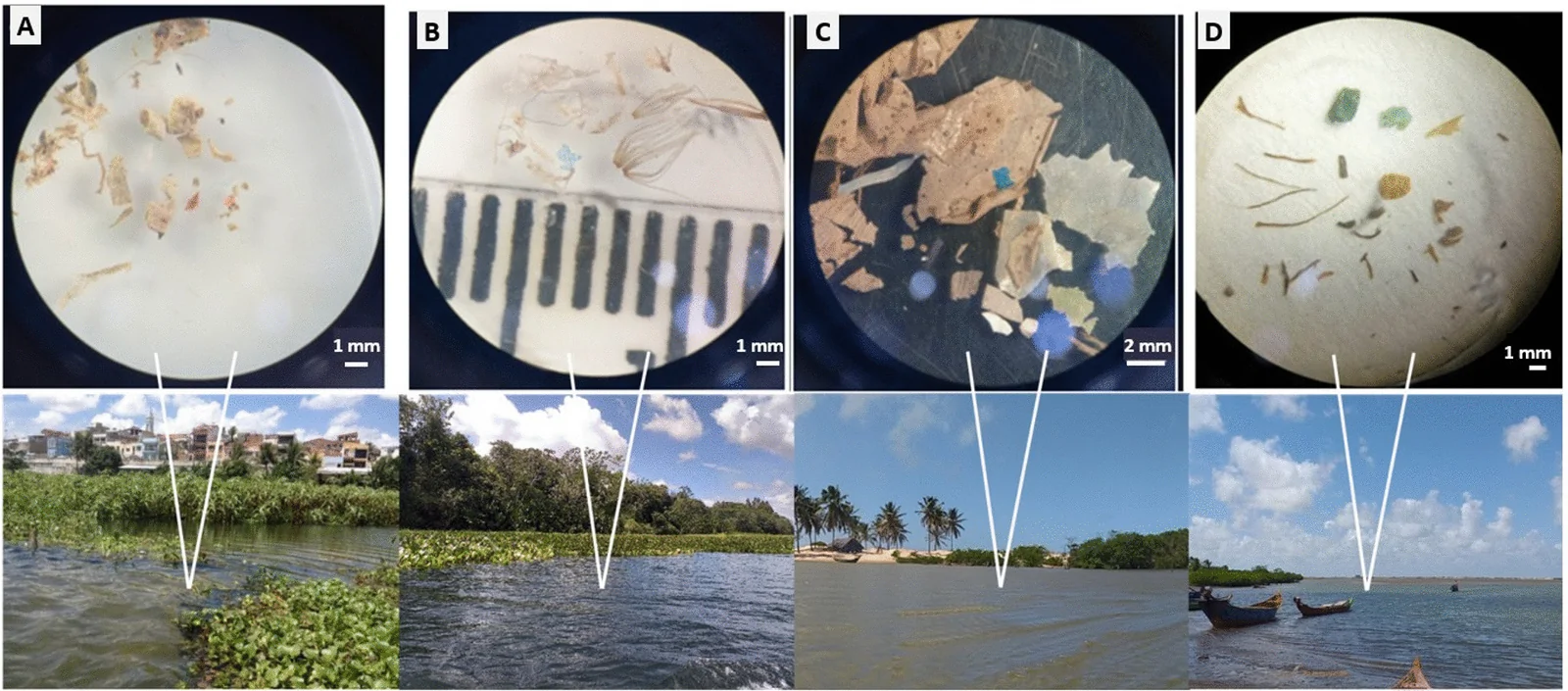
Example of possible microplastics found in the samples. **A** São Francisco River – Propriá; **B** São Francisco River – Santana de São Francisco; **C** Parapuca River – Brejo Grande; **D** Parapuca River – Pacatuba

In all collections, white/aged plastics were predominant in microplastics (Fig. 3), representing more than 90% of the total identified, which may indicate degradation due to sun exposure and aging of plastics. Regarding morphology, the most common shapes were fragments, followed by fibers/filaments, which points to secondary fragmentation processes of larger plastics.

The abundance of microplastics was also determined in the study conducted in Australia by Reisser et al. (2013), where 813 items were identified, 84.7% of which were white/transparent. Pan et al. (2019) conducted a study in the Pacific Northwest and obtained an abundance of 10,000 items/km^2^ and also reported the predominance of white among microplastics present in aquatic environments (57.4%). Tien et al. (2020) used a more robust methodology and larger sampling volumes, which enabled an even more detailed analysis of the presence of microplastics with an abundance of 334–1058 items/m^3^ in water samples.

It is important to highlight that the presence of microplastics in rivers, lakes, and oceans results in other associated problems, since there is also the possibility of these microplastics accumulating in bivalve mollusks, such as oysters, mussels, and clams, which are commonly produced in aquaculture for human consumption, leading to concerns about the quality and safety of the product that reaches consumers (Bebianno et al., 2025; Jankauskas et al., 2024).

Microplastic studies are important for assessing risks and impacts and improving monitoring of this pollutant in the aquatic environment, in order to contribute to public awareness, environmental planning, and coastal zone management processes.

#### Microplastics in beach sand

Microplastics were collected from sandy sediments on the beaches of Ponta dos Mangues, in the municipality of Pacatuba, and Carapitanga, in Brejo Grande. These stretches of sand are located between the Parapuca River and the Atlantic Ocean, forming a transition zone between riverine and marine environments.

In Pacatuba, collections took place on three different dates. In the sample collected on June 20, 2018, the mass of microplastics was 0.2004 g, with an estimated density of 10.8 items/m^2^. On May 21, 2019, an increase was observed, with a mass of 0.2547 g and an estimated 22.8 items/m^2^. In the collection on December 16, 2019, the recorded mass was 0.1398 g, while the estimated number of items was 20.4 per square meter.

In Brejo Grande, due to logistical limitations and the natural characteristics of the location, it was only possible to collect two samples. The sample collected on February 1, 2019, had a microplastic mass of 0.1106 g and an estimated density of 9 items/m^2^. The collection carried out on August 6, 2019, at the same location revealed a higher mass of 0.4001 g, with an estimated density of 20.8 items/m^2^.

It should be noted that the number of items per square meter was estimated based on a count in ten 50 × 50 cm quadrants, totaling an area of 2.5 m^2^. The term “estimated number of items per square meter” rather than “exact number of items” is due to the fragmentation of some particles, which makes it difficult to accurately count microplastics (Giardino et al., 2022).

**Fig. 4:**
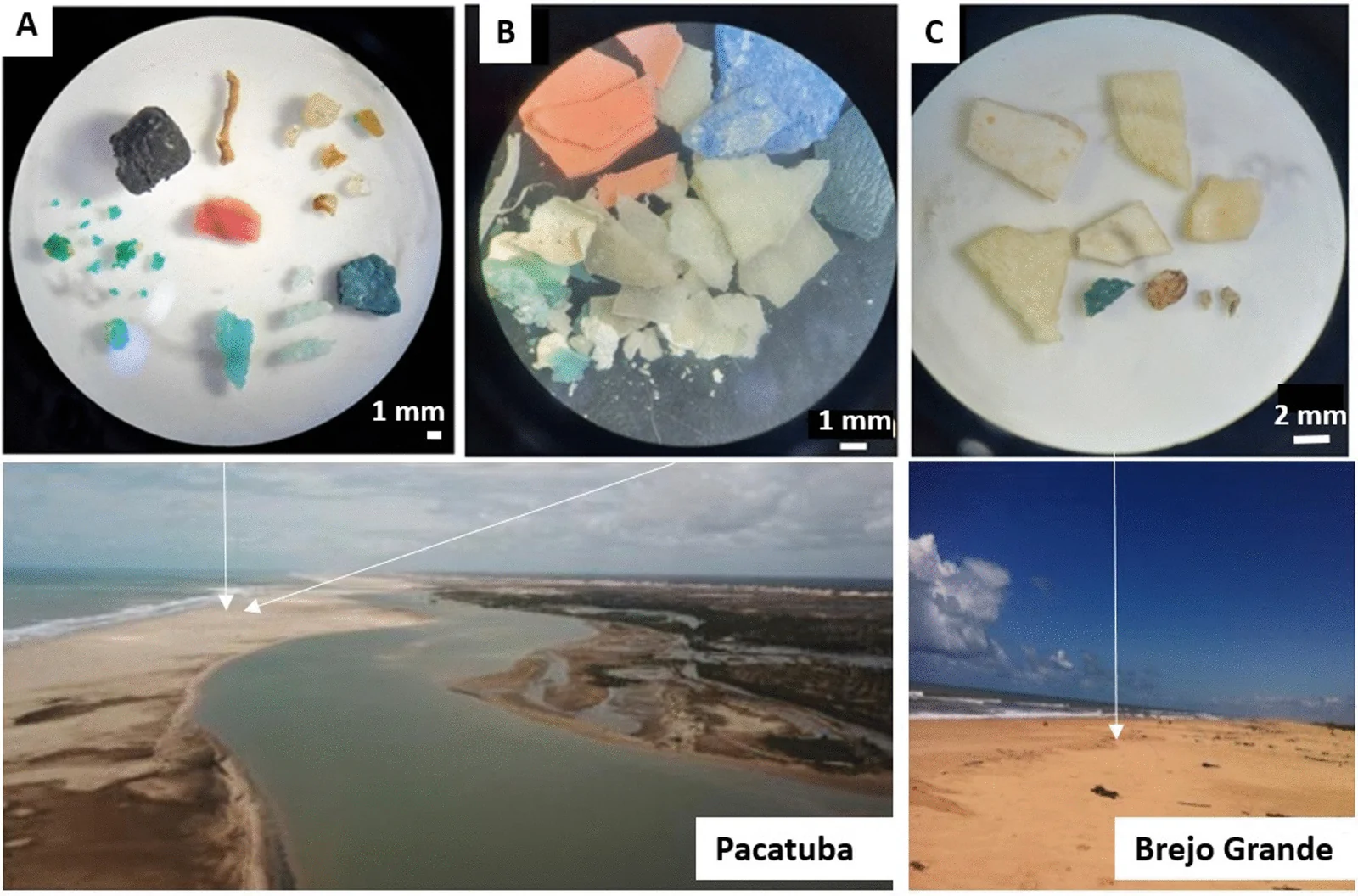
Examples of possible microplastics extracted from sand samples between 2018 and 2019; **A** Ponta dos Mangues Beach—June 20, 2018 (Pacatuba, Sergipe); **B** Ponta dos Mangues Beach—May 21, 2019 (Pacatuba, SE); **C** Carapitanga Beach—February 1, 2019 (Brejo Grande, SE)

Figure 4 shows representative examples of possible microplastics found in sandy sediments collected in the study area, around a conservation unit. The presence of these particles in the Santa Isabel Biological Reserve area highlights the wide dispersion of these contaminants, even in protected environments.

The identified particles presented varied morphologies, predominantly fragments, and in terms of color, the white/aged class prevailed, followed by green and blue. In the work of Phuong et al. (2018), the predominant colors were gray (60%) and white (13%), with a total of eight colors observed: gray, red, white, green, black, blue, pink, and yellow.

Several studies worldwide have investigated the concentration of microplastics in beach sediments, showing variation in observed values depending on the region and methodology used (Hidalgo-Ruz & Thiel, 2013; Lots et al., 2017; Graca et al., 2017; Phuong et al., 2018; Yaranal et al., 2021). These variations reflect not only differences in the environment and coastal use, but also the use of different units of measurement to express their results (items per m^2^, items per kg, or mg per kg), which makes direct comparisons between studies difficult.

A study conducted on sediments in the Bay of Biscay, France, investigated microplastic (MP) contamination and identified an average of 67 (± 76) MPs per kilogram of dry sediment (Phuong et al., 2018). To facilitate comparison with the present study, we also calculated the average number of items/kg, which corresponded to 58 items/kg of dry sediment for Brejo Grande and 74 items/kg of dry sediment for Pacatuba.

In Chile, concentrations ranged from 1 to 805 items/m^2^ (Hidalgo-Ruz & Thiel, 2013). In the Brazilian context, Carvalho and Baptista Neto (2016) analyzed sediments from Guanabara Bay, reporting concentrations between 12 and 1,300 items/m^2^. The study by Maynard et al. (2021) assessed the occurrence of microplastics on six beaches along the Brazilian coast, including Viral and Ponta dos Mangues beaches in Sergipe. The highest quantities were recorded at Viral beach (30.4 items/m^2^) and Ponta dos Mangues beach (17.4 items/m^2^).

### Inventory of macroplastics in the sand of the São Francisco River estuary beach

**Fig. 5:**
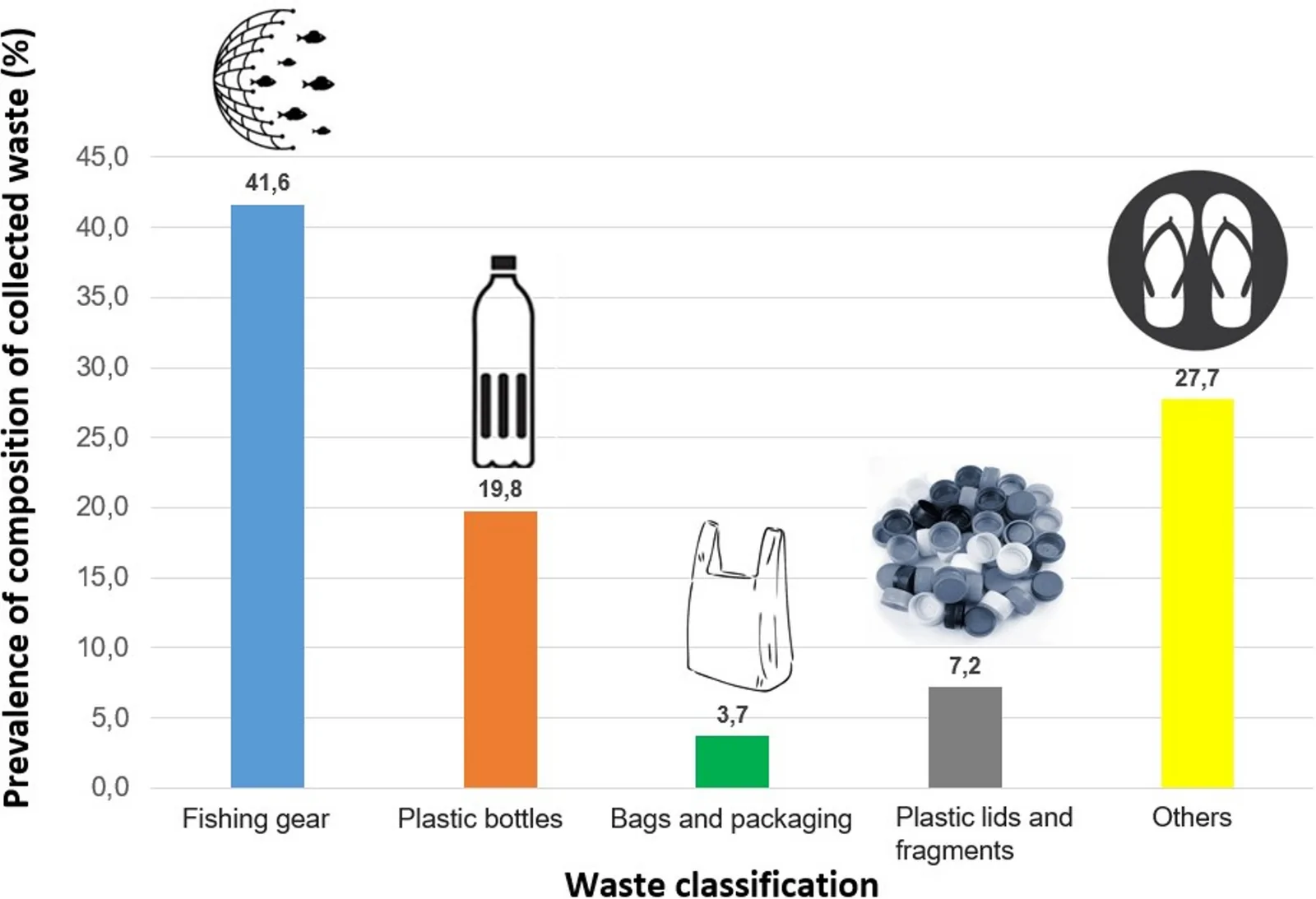
Graph showing the prevalence of macroplastic classes collected in Pacatuba and Brejo Grande between 2018 and 2019

Considering that the main source of microplastics detected in the São Francisco River estuary is associated with the fragmentation of larger plastic items, macroplastics were collected at two representative points along the coast: Ponta dos Mangues (Pacatuba, Sergipe) and Carapitanga (Brejo Grande, Sergipe). The sampling, carried out between 2018 and 2019, revealed variations in both the total mass of plastic waste (g) and its typological composition (g), including fishing gear, bottles, plastic bags and packaging, lids/fragments, and others. Furthermore, the prevalence of the typological composition (%) was calculated according to the methodology and can be seen in Fig. 5.

In Pacatuba, the total amount of material collected increased throughout the study period, with a sharp peak in December 2019, when 2457 g were recorded. The composition was dominated by fishing gear (1298 g), followed by bottles (416 g), bags (60 g), caps and fragments (90 g), and other plastic waste (593 g). In contrast, June 2018 showed the lowest total mass (36.2 g), composed mainly of bottles (15.5 g) and bags/packaging (8.2 g), in addition to fishing gear (7.1 g) and caps and fragments (5.4 g).

In Brejo Grande, the highest mass was recorded in February 2019 (551 g), predominantly composed of plastic bottles (214 g), fishing gear (42 g), bags and packaging (19 g), caps and fragments (84 g), and other materials (192 g). The lowest value was observed in August 2019 (219 g), with a predominance of caps and fragments (56 g) and other waste (119 g), followed by bags and packaging (33 g) and fishing gear (11 g).

It was observed that in Pacatuba there was an upward trend in the amount of macroplastics collected from the beach sand. In Brejo Grande, on Carapitanga beach, however, a decrease was observed. One of the factors that may explain this decrease is the advancing tide observed on the beach and the burial of the waste deposited there.

In addition, photographs were taken of the collected material, highlighting fishing gear, chemical packaging (resin, oil, etc.), and plastic caps and fragments. Among the bottles and their labels, one bottle originated from China, two from India, and one water bottle label from Malaysia. The bottles of international origin were weighed, totaling 75 g, and their prevalence was calculated relative to the total mass of waste collected in the study area (4723.2 g), representing 1.5% of the inventoried waste.

The presence of plastic water bottles of international origin on Pacatuba beach may be associated with improper disposal by vessels, given the beach’s proximity to the Port of Sergipe and major shipping routes. Recent evidence indicates that illegal dumping of plastic bottles from ships is responsible for much of this type of waste in coastal areas close to shipping routes, exceeding the contribution from land-based sources, reflecting shortcomings in maritime environmental monitoring and regulatory enforcement (Ryan, 2023; Ryan et al., 2019, 2021).

Monteiro et al. (2024) assessed the presence of plastic waste on five beaches in the city of Aracaju, Sergipe, Brazil. In this study, 22,298 g of plastic were collected from the beaches studied, with Praia do Viral standing out with 4382 g per 100 m. In addition, this study compared the amount of plastic on the sandy strip and in the vegetation strip + (sandbank) where much of the waste is retained.

Gárces-Ordóñez et al. (2020) assessed the presence of plastic waste on 43 sandy beaches in Colombia. The amounts of macroplastic ranged from 35 ± 15 to 81 ± 23 items per 100 m. According to the authors, plastic pollution is a global problem that poses a threat to coastal and marine ecosystems and community livelihoods, and requires effective solutions that are adaptable to local conditions.

Collecting data on beach litter provides information on quantities, trends, and sources of this litter. This information can be used to develop effective mitigation measures and also to assess the effectiveness of existing legislation and regulations (OSPAR, 2010).

Plastics and plastic fragments account for most of the debris polluting estuarine beaches. Most of these fragments come from items discarded within the estuary or river basin. The case of fishing-related items illustrates well how a large item used to its limit and improperly discarded can fragment into pieces small enough to threaten biota in different ways (entanglement and then ingestion). Therefore, regardless of size and type, impacts on aquatic biota and human populations arise when plastics are discarded into the environment (Ivar do Sul e Costa, 2013).

### Integrated environmental assessment

The highest values for plastic pollution markers (BPA and DBP) and microplastics in surface water were observed in the municipality of Propriá. These values may be related to the impacts of urbanization in this region—the discharge of domestic effluents into the São Francisco River and the improper disposal of plastic waste that can release these substances into the environment. In this context, surface water may act as an important transport pathway for plastic particles and associated chemical additives throughout the estuarine system. In addition, the São Francisco River carries a load from its entire course and all the activities that are carried out along the way. The occurrence of microplastics in surface waters may also reflect fragmentation processes of larger plastic debris during river transport.

The highest concentrations of microplastics and macroplastics in the sand were found in the municipality of Pacatuba. These findings suggest that sandy sediments may function as zones of deposition and accumulation of plastic debris within the estuary. The origin of this waste may be maritime (intentional or unintentional), from vessels passing through the region due to the Port of Sergipe, or terrestrial, from improper waste disposal. The predominance of macroplastics in this area may indicate local retention processes, while the presence of microplastics may be associated with the progressive fragmentation of deposited materials**.** The sampling sites Brejo Grande and Pacatuba, despite being located in a Federal Conservation Unit, the Santa Isabel Biological Reserve, and not having urbanization on the banks of the river as intense as the city of Propriá, have fishing, shrimp farming, agriculture, and tourism as activities that can also generate negative impacts on the environment**.** These results reinforce that protected areas are not isolated from regional plastic contamination dynamics.

**Fig. 6:**
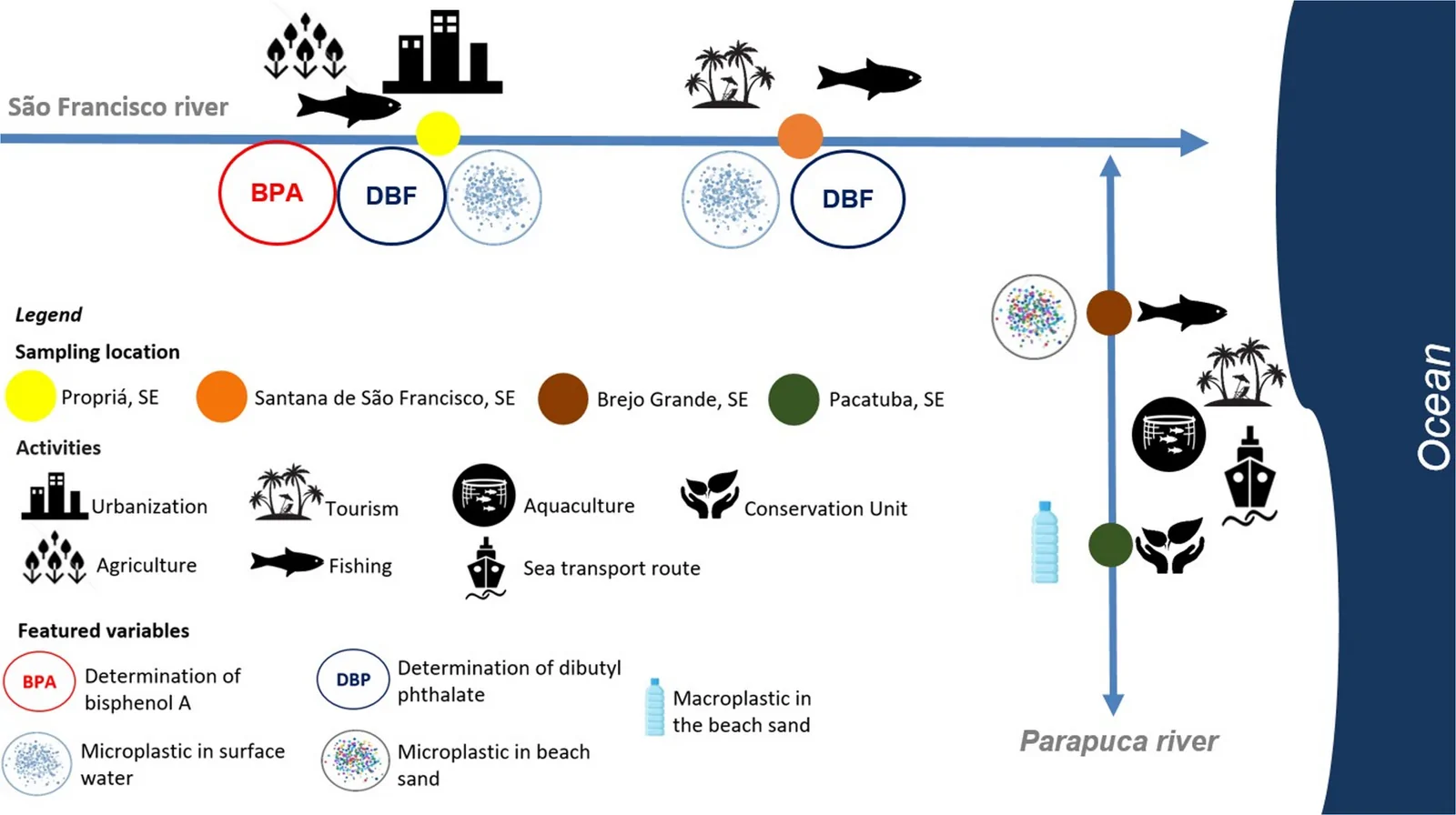
Graphical representation of the variables that stood out in the study stations

Figure 6 is a graphical representation of the summary of the main results obtained at the analysis stations in this study. The figure integrates the spatial distribution of macroplastics, microplastics, and plasticizers across the studied environmental matrices. Where, as shown by the larger arrow, the São Francisco River passes through the cities of Propriá (yellow) and Santana de São Francisco (orange) and meets the tributary, the Parapuca River (smaller arrow), which passes through Brejo Grande (green) and Pacatuba (brown), both of which meet the Atlantic Ocean. All details of the icons can be found in the legend.

Therefore, this study represents a benchmark multicompartmental environmental assessment, and future investigations with larger sample sizes could incorporate multivariate and statistical correlation analyses to better explore the interactions between the matrices.

### Study limitations and future research directions

The present study focused primarily on the quantification, spatial distribution, and characterization of plastic debris based on morphological attributes, as well as the determination of plastic-associated contaminants (BPA and DBP) in the São Francisco River estuary. Therefore, the identification of polymer composition was beyond the scope of the research objectives.

A limitation of this study is that microplastics were classified according to visual characteristics, including shape, color, and size, without confirmation of polymer composition through spectroscopic techniques such as Fourier Transform Infrared Spectroscopy (FTIR) or Raman spectroscopy. Consequently, the results should be interpreted as estimates of plastic particle abundance and distribution rather than a detailed characterization of polymer types.

Future studies should incorporate polymer identification techniques, such as FTIR, Raman spectroscopy, or pyrolysis-GC/MS, to confirm particle composition and improve source-tracking analyses. Additional investigations may also evaluate the relationships between specific polymer types, additive release, degradation processes, and contaminant transport in estuarine ecosystems, contributing to a more detailed understanding of the environmental fate of plastic pollution in tropical estuaries.

## Conclusion

The analysis of plastic pollution markers with endocrine disrupting potential identified the presence of bisphenol A in 100% of the samples analyzed and dibutyl phthalate in 67%, highlighting the influence of anthropogenic contamination in the waters of the São Francisco River and its tributary, the Parapuca.

The quantification and characterization of plastic pollution markers, as well as micro and macroplastics in the estuarine region, especially in the vicinity of a Conservation Unit, highlight the vulnerability of these ecosystems, which act as the final recipients of all the cargo transported along the river basin to the sea.

Thus, it can be concluded that the methodological integration adopted in this interdisciplinary assessment of the São Francisco River estuary enables a simultaneous and detailed analysis of different forms of environmental pollution. This integrated approach contributes to a more comprehensive understanding of issues that are still at early stages of investigation within the river basin, such as the occurrence of microplastics and associated chemical compounds (BPA and DBP). The results obtained provide relevant support for the formulation of public policies, promote environmental awareness within the local community, and contribute to evidence-based decision-making grounded in scientific data.

Plastic materials play an important role in various applications, especially in the healthcare sector, including hospital supplies, hygiene products, and protective equipment. However, whenever possible, their replacement with more sustainable alternatives should be promoted, along with conscious consumption and proper disposal and recycling practices. Strengthening these actions contributes to a circular economy, reduces environmental impacts, and promotes more sustainable plastic management.

## Data Availability

The authors declare that the data supporting the findings of this study are available within the article. If raw data files in another format are required, they are available from the corresponding author upon reasonable request. The source data are provided with this article.
